# Repeated evolution of convergent iridescence in closely related species of *Morpho* butterflies living in sympatry

**DOI:** 10.1093/evlett/qrag028

**Published:** 2026-07-10

**Authors:** Joséphine Ledamoisel, Vincent Debat, Violaine Llaurens

**Affiliations:** Centre Interdisciplinaire de Recherche en Biologie, Collège de France, Paris, France; Institut de Systématique, Evolution et Biodiversité, Muséum National d’Histoire Naturelle, Paris, France; Centre Interdisciplinaire de Recherche en Biologie, Collège de France, Paris, France; Institut de Systématique, Evolution et Biodiversité, Muséum National d’Histoire Naturelle, Paris, France; Centre Interdisciplinaire de Recherche en Biologie, Collège de France, Paris, France

**Keywords:** evasive mimicry, phenotypic evolution, ecological interactions, iridescence, sympatry, insects

## Abstract

The evolution of visual traits in closely related species living in sympatry is highly influenced by their ecological interactions: while sexual selection tends to promote the divergence of visual cues involved in mate choice, natural selection via predation may promote the convergence of dissuasive signals between prey species, especially in unpalatable or evasive prey. Here, we investigate the impact of sympatry on the evolution of the blue structural coloration in the wings of two closely related *Morpho* butterfly species across several localities throughout Central and South America. Dorsal iridescence might affect mate choice and species recognition, which should promote its local divergence among species. However, the bright flashes and dynamic color patterns produced by iridescence during flight might also increase survival by confusing predators and favoring escape. Such an effect might in turn lead to convergence in wing iridescence between evasive species occurring in sympatry, a phenomenon dubbed evasive mimicry. To test the effect of these putative antagonistic selective forces on visual cues evolution, we quantified the variation of the structural blue color displayed at 13 different combinations of illumination/observation angles, on the wings of 2 closely related *Morpho* species. We contrasted 10 sympatric and 11 allopatric locations and specifically compared the phenotypic distances between individuals from different species. Phenotypic distances between heterospecific pairs of individuals were significantly smaller in sympatry, suggesting local convergences of iridescence consistent with the expected pattern assuming evasive mimicry. Interestingly, sexual dimorphism was found between males and females, suggesting that the trade-off between natural and sexual selection on the evolution of iridescence might differ between sexes. Our results suggest that local predation pressures may promote repeated evolutionary convergence of structural coloration between evasive prey species living in sympatry.

## Introduction

How do ecological interactions shape trait evolution in closely related species occurring in sympatry? Closely related species share a common phylogenetic history and are thus more likely to present similar suites of traits (Blomberg et al., 2003) favoring their co-existence within the same habitat (Burns & Strauss, 2011). However, niche overlap caused by their phenotypic proximity might trigger acute competition for resources or reproduction, promoting the divergent evolution of traits involved in foraging and mate recognition in sympatry (Pfennig & Pfennig, 2009). In particular, in animals where visual cues are involved in mate recognition, divergent evolution of visual traits among closely related species is frequently observed in sympatry (Allen et al., 2014; Stanger-Hall & Lloyd, 2015). Yet, the evolution of visual traits in closely related prey species living in sympatry might also depend on the perception and behavior of shared predators (Anderson & de Jager, 2020; Nosil & Crespi, 2006). Predation pressures can indeed promote either directional or diversifying selection acting on visual traits displayed in prey (Nokelainen et al., 2024). Shared predation pressures may for instance promote the evolution of mimicry among unprofitable prey species living in sympatry, making the evolution of visual traits in these different prey species nonindependent from one another (Puissant et al., 2023). Comparing the evolution of visual traits in closely related species in sympatric *vs*. allopatric areas thus allows to investigate the relative contributions of shared selective pressures and species interactions on the evolution of traits in sympatry.

The nonindependent evolution of coloration between closely related species living in sympatry is documented in a large number of animal taxa. The evolution of coloration has been shown to be strongly influenced by selection exerted by local abiotic factors such as temperature (Clusella Trullas et al., 2007), humidity (Delhey, 2019), or UV radiation (Bastide et al., 2014), resulting in similar coloration among sympatric species because of these shared selective pressures. Phenotypic evolution of closely related species may also be deeply impacted in sympatry by natural selection arising from ecological interactions both (1) among those closely related species and (2) between these and other species from other guilds. Indeed, coloration is often involved in competitive interactions, mate recognition, and mate choice, as well as predator deterrence or avoidance (Protas & Patel, 2008), generating antagonistic selection regimes acting on the evolution of coloration in closely related species in sympatry. Reproductive interference caused by the poor fitness of hybrids produced by crosses happening between visually similar species (Gröning & Hochkirch, 2008) might promote divergence in coloration: for example, larger divergence in throat fan colorations has been documented among closely related species of lizards in sympatric areas *vs*. allopatric areas (Lambert et al., 2013). In contrast, predation pressures shared by different prey species in sympatry have been shown to lead to convergent evolution of coloration in multiple taxa (Joron & Mallet, 1998; Puissant et al., 2023). Unpalatable prey indeed frequently display conspicuous visual signals (Caro & Ruxton, 2019) recognized and avoided by predators (Darst & Cummings, 2006; Ihalainen et al., 2007), therefore generating a positive density-dependent selection acting on visual trait evolution both within and among unpalatable prey species (Joron & Mallet, 1998). Mimicry caused by predator learning has also been hypothesized to happen in palatable prey with high escape abilities (Pinheiro, 1996; Ruxton et al., 2004; Young, 1971). Behavioral experiments have indeed shown that birds that were repeatedly presented different types of colored prey quickly stopped to attack evasive prey associated with specific conspicuous coloration; the association between evasiveness and coloration was even more rapidly learnt than when the same coloration was associated with unpalatability (Páez et al., 2021). This learning process could eventually lead to the evolution of similar conspicuous colorations associated with evasiveness (i.e., evasive mimicry, Linke et al., 2022; Ruxton et al., 2004), among individuals belonging to different species submitted to the same predator community. For example, beetles sharing similar colored traits associated with evasive flight are less targeted by visual predators, suggesting that resembling to a *hard-to-catch* prey may improve survival (Guerra et al., 2024). Convergent evolution of coloration might thus be promoted in closely related species living in sympatry when they exhibit high evasiveness. Iridescence (i.e., a structural coloration varying depending on illumination/observation angles) is a particularly relevant trait to investigate the balance between natural and sexual selection acting on trait convergence *vs*. divergence in closely related species occurring in sympatry because (1) the selective trade-off might results in visual convergence at certain angles and divergence in others, making iridescence a relevant species recognition cue (e.g., in hummingbirds, Gruson et al., 2021), and (2) iridescence can strongly contribute to flash coloration, therefore directly enhancing evasive capacities (Murali, 2018).

Here, we thus investigate variations in iridescent colors within and among two closely related species of *Morpho* butterflies living in sympatry, using evasive mimicry as a working hypothesis. We specifically focus on the sister species *Morpho helenor* (Cramer, 1776) and *Morpho achilles* (Linnaeus, 1758). These neotropical species display large overlapping geographic distributions (Blandin & Purser, 2013), allowing to estimate the effect of ecological interactions on the evolution of their traits by comparing multiple sympatric and allopatric localities. They are characterized by their very similar conspicuous dorsal wing patterns composed of a bright blue band, generated by specific scale nanostructures (Giraldo et al., 2016), surrounded by a dark pigmentation enhancing contrast. The blue dorsal coloration varies depending on the angle of observation and illumination (Ledamoisel et al., 2025), thereby producing a dynamic iridescent visual phenotype during flapping flight. Male mate choice experiments using female wing models in the wild (Le Roy et al., 2021) and in controlled conditions (Ledamoisel et al., 2025) suggested that iridescent coloration may be involved in mate localization and mate choice in these *Morpho* species. Interestingly, the ventral wing surface exhibits a dull brown pigmentary coloration contrasting with the bright dorsal coloration. The blue iridescence, coupled with their erratic flapping flight, thus generates a confusing visual signal: the alternation between the ventral matte and the dorsal bright blue sides of their wings during flight creates conspicuous flashes. Combined with their unpredictable flight trajectories, these flashes could make these butterflies very difficult to follow, locate, and catch by bird predators (Young, 1971); Capture-Mark-Recapture experiments indeed showed that iridescent flashes were as efficient as cryptic coloration in predation avoidance (Vieira-Silva et al., 2024). In turn, these very conspicuous signals could make them highly memorable to predators, which would learn to refrain from attacking such hard-to-catch blue prey. In agreement with this hypothesis, observations in the field have shown that *Morpho* butterflies are often sight-rejected by wild insectivorous birds, as compared to alternative butterfly prey from the same community (Pinheiro & Campos, 2019). Overall, the evasive capacities of *Morpho* butterflies combined with their very conspicuous iridescent coloration could provide protection against predators, and promote the evolution of similar iridescent patterns in different species facing the same predator community (i.e., evasive mimicry). Because chemical defenses have never been reported in those butterflies, and because their caterpillars feed on nontoxic Fabacea and bamboo hostplants (Blandin et al., 2014; Chazot et al., 2021), evasiveness rather than toxicity has been assumed to promote color pattern similarity between sympatric species of *Morpho*. A previous study investigating variations in the width of the dorsal blue band in *M. helenor* and *M. achilles* indeed found that these species share more similar wing *patterns* when they live in sympatry compared to allopatry (Llaurens et al., 2021), consistent with the hypothesis of local convergence of wing pattern expected via evasive mimicry. However, unlike pigmentary aposematic signals, iridescent coloration may provide a direct survival benefit by enhancing prey escape independently of predator learning, thereby reducing the additional selective advantage of mimetic resemblance and potentially limiting convergence among sympatric species. Divergence in reflectance at certain angles might provide relevant species recognition cue while maintaining a flash effect limiting predation. The common selective trade-off acting on different visual cues displayed in these closely related species might result in different outcomes for iridescent properties of the blue patches as compared to color pattern. Comparing color pattern and iridescence evolution may provide a more comprehensive understanding of the mechanisms shaping the evolution of visual cues in these butterflies.

We used museum specimens to quantify iridescence variation within and between the closely related species *M. helenor* and *M. achilles* in both allopatric and sympatric ranges. We computed the phenotypic distances based on iridescence between heterospecific pairs of individuals across localities and tested whether these distances were lower among pairs sampled in the same locality compared to pairs collected in different localities. We then used visual modeling to test whether birds are able to perceive the differences in iridescence among species within localities. Altogether, our study aims at testing whether geographic variation in iridescent blue displayed in closely related *Morpho* species is consistent with the pattern expected if evasive mimicry shapes the evolution of coloration in sympatry.

## MATERIALS AND METHODS

### Sampling zones and museum specimens

We sampled *Morpho* specimens stored in the Muséum National d’Histoire Naturelle (MNHN) in Paris (France). *Morpho* butterflies are distributed across Central and South America, from Mexico to North Argentina (Blandin & Purser, 2013). *Morpho helenor* contains 40 described subspecies with substantial wing color pattern differences, and has the largest distribution out of the two species studied here, with geographic overlap with *M. achilles* throughout the Amazonian basin (Blandin, 2007) . *Morpho helenor* thus sometimes co-exists with *M. achilles* in sympatry in the same microhabitat (the forest understory) at the same period of the year, but is also found isolated in allopatric regions located in Mexico, Costa Rica, Argentina, Eastern Brazil, and also in some localities in Western Ecuador, Northern Peru, and Venezuela (see Figure 1).

**Figure 1.**
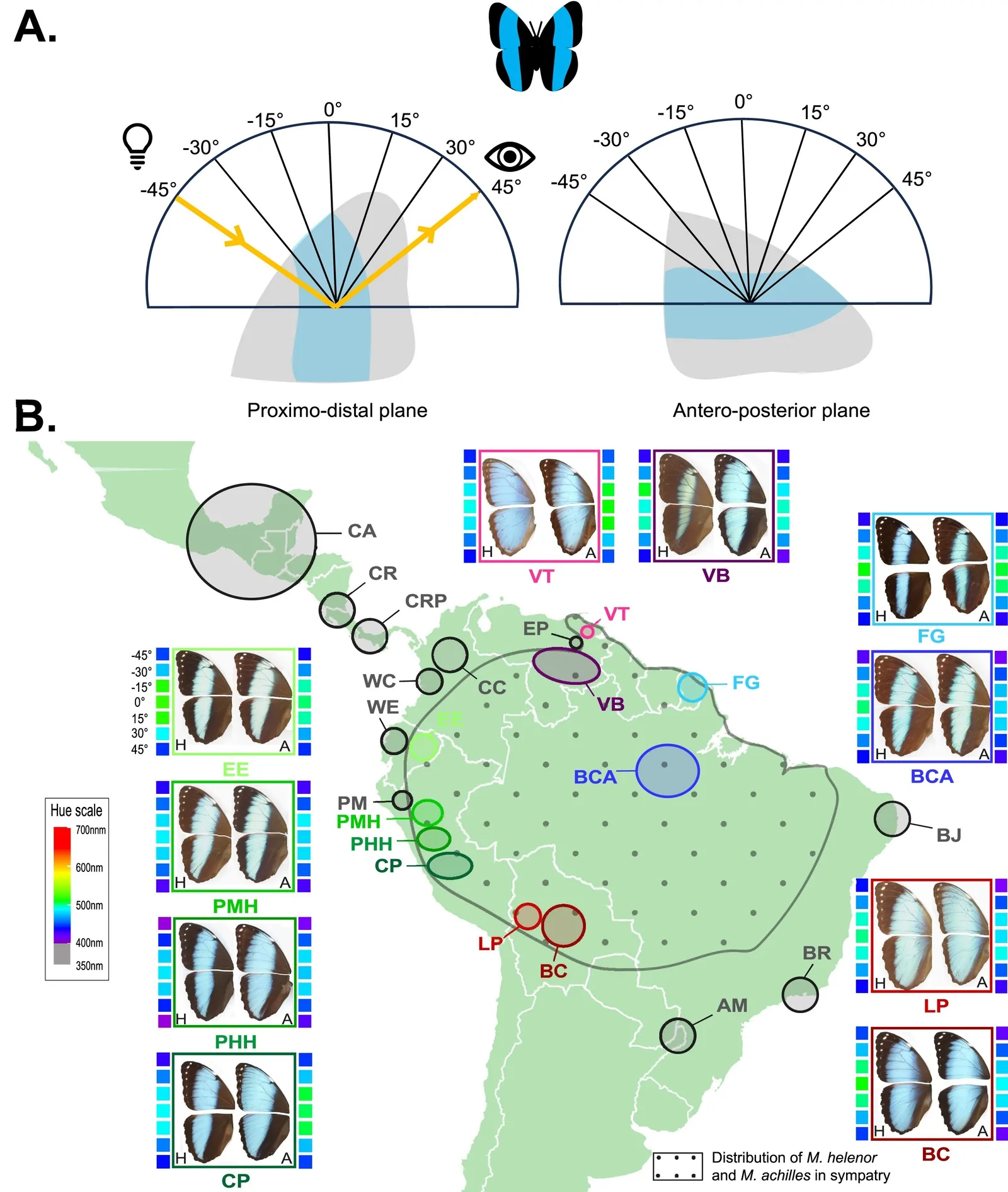
(A) Iridescence measurement protocol and (B) geographic distribution of the *Morpho* samples studied, partitioned into 21 localities located across Central and South America, and their mean variation of coloration at different angles of illumination. (A) The specular reflectance of *Morpho* wings was measured when illuminating the wing along two planes (proximodistal and anteroposterior planes) at seven angles of illumination each, resulting in 13 reflectance spectra (the normal being shared by the two planes). Yellow arrowed lines show the position of illumination and observation fibers for a specular measurement of the reflectance of a wing in the proximodistal plane at a 45° angle as an example. (B) The black areas highlight the localities where *M. helenor* lives in allopatry from its closely related species *M. achilles* (CA = Central America, CR = Costa Rica, CRP = Costa Rica Panama, CC = Central Columbia, WC = Western Columbia, WE = Western Ecuador, PM = Peru Maranon, AM = Argentina Missiones, BR = Brazil Rio de Janeiro, BJ = Brazil Joao, EP = Venezuela El Pao). The colored areas indicate the localities where *M. helenor* lives in sympatry with *M. achilles*, either west (shades of green: EE = Eastern Ecuador, PMH = Peru Middle Huallaga, PHH = Peru High Huallaga, CP = Central Peru), south (shades of red: LP = Bolivia La Paz, BC= Bolivia Cochabamba), east (shades of blue: BCA = Brazil Central Amazon, FG = French Guiana), or north (shades of pink: VB = Venezuela Bolivar, VT = Venezuela Tucupita) of the Amazon basin. The pictures show the wing morphology of male *M. helenor* (H, on the left) and *M. achilles* (A, on the right) depending on the locality. Their respective hue variation measured along the proximodistal plane of their wings is pictured next to each subspecies: from top to bottom, each square represents the mean hue measured when illuminating the wings every 15° from −45° to 45°. Note that the entire hue variation at every angle of illumination measured on both wing planes, for each subspecies, sex, in every locality (allopatry or sympatry), can be found in Figure S1. This hue representation follows an RGB scale: it is only illustrative and does not simulate the actual vision of neither butterflies nor predators.

Sampling zones were defined referring to *M. helenor* subspecies (Blandin, 2007; Le Moult & Réal 1962) and details on the delimitation of these subspecies can be found in the supplementary material of Llaurens et al. (2021). We used specimens collected in 21 localities across Central and South America, covering 18 *M. helenor* subspecies: subspecies distributed across large geographical zones were divided into different localities. Among those 21 localities, 10 are defined as *sympatric*. The remaining 11 localities are defined as *allopatric* for *M. helenor* because *M. achilles* does not occur in those regions (Gayman et al., 2016; Llaurens et al., 2021). We quantified the variations in iridescence of *n* = 351 specimens in total, including *n* = 249 *M. helenor* and *n* = 102 *M. achilles* butterflies and used both males (*n* = 201) and females (*n* = 150). Females are much rarer than males in collections because of behavioral differences between sexes: *Morpho* males are more often observed flying along forest trails as compared to females and are thus more often caught by humans (see Table S1 for the detailed sampling plan).

### Iridescence measurement protocol

Iridescence is defined as the variation of coloration of a surface depending on the angle of illumination and/or the angle of observation. The reflectance of *Morpho* wings was thus measured at different angles of illumination and observation using a specular reflection set-up (i.e., by positioning the lightening and receiving probes symmetrically relative to the normal to the wing surface), following the protocol described in Ledamoisel et al. (2025). Briefly, wings were positioned on a flat surface, following a template to ensure that their orientation remained the same across all measurements. Iridescence was measured in the wing area delimited by the M3 and Cu1 veins using a spectrophotometer (AvaSpec-ULS2048CL-EVO-RS 200–1100 nm, Avantes) coupled with a deuterium halogen light source (AvaLight-DH-S-BAL, Avantes), and two optical fibers (FCR-7UVIR200-2-1.5 × 100 and FC-UVIR200-2-1.5 × 100, Avantes). The angular position of the two fibers was precisely controlled using an angled fiber holder (AFH-15, Avantes). Reflectance was measured every 15° in the proximodistal plane of the wings, the normal to the wing being set at 0°, resulting in seven angular positions (−45°, −30°, −15°, 0°, 15°, 30°, and 45°). The same protocol was repeated in the anteroposterior plane, resulting in a total of 13 reflectance spectra ranging from 300 to 700 nm, measured at 13 angular positions per individual (the normal being common to the two plans, see Figure 1A).

Although the repeatability of this protocol was validated in Ledamoisel et al. (2025), we repeated each measurement three times at every angle of illumination for 10 individuals. Repeatability was tested using the R package rptR 0.9.22 (Stoffel et al., 2017): no significant difference was detected between replicates for any of the tested angles (Table S2).

### Spectral data analysis and statistics using colorimetric variables

#### Iridescence description

We used the R package pavo2 2.9.0 (Maia et al., 2019) on each of the 13 reflectance spectra measured per individual (*n* = 351) to compute the three colorimetric variables most commonly used to describe visual signals: hue (i.e., the wavelength corresponding to the maximum reflectance, describing color), brightness (i.e., the mean reflectance over a spectrum, describing the amount of reflected light), and chroma (i.e., the ratio of the reflectance range of each spectra divided by the brightness, describing color saturation). To focus on the variation of blue iridescence, we extracted these colorimetric variables on the (350–700 nm) range for each spectrum. Because the normality and heteroscedasticity assumptions were rarely met for the distribution of values in these three parameters, we used permutation-based ANOVAs (vegan 2.6.4 package, Oksanen et al., 2001) to test the effect of sex, species, angle of measurement, and the interactions between those variables on each wing plane separately and within each locality.

To investigate the variation of iridescence among individuals, we performed a principal component analysis (PCA) based on hue, brightness, and chroma from the 13 reflectance spectra per individual. Each dot in the PCA space thus corresponds to the summary of reflectance spectra at different angles of an individual wing: the closer the points, the more similar the iridescence between individuals. The first 10 PCs (representing more than 98% of the explained variance) were retained and used in PERMANOVA to test for the effect of species, sex, and locality on iridescent patterns. We also tested for the potential effect of wing degradation associated with time spent in the museum collections since the sampling date (Supplementary Information 1) and found no significant effect of the sampling date on iridescence variation.

#### Testing iridescence convergence

To test whether individuals from either species were more similar within each sampling zone than expected by chance, we used the permutation-based approach previously described in Llaurens et al. (2021). The phenotypic resemblance between species was computed as the average Euclidean distances between individuals across species and localities, in the PCA space defined by the first 10 PCs computed from the hue, brightness, and chroma variations across angles. To test whether these phenotypic distances between individuals from different species are smaller when individuals are sampled within the same geographic areas as compared to randomly sampled individuals, a null distribution of phenotypic distance was built by randomly shuffling the location of the different individuals. For 10,000 permutations, the average phenotypic distance between interspecific pairs of *M. helenor* and *M. achilles* individuals was computed. A null distribution of phenotypic distances was thus generated assuming that geographic variation in color pattern evolved independently in *M. helenor* and *M. achilles* found in sympatry and allopatry. The significance of the phenotypic convergence among species in sympatry was assessed by comparing the observed average distance between interspecific pairs of individuals in sympatry to this null distribution: an observed distance was considered significantly smaller (i.e., greater phenotypic resemblance) when it fell below 95% of the randomized values. Because individuals from allopatric populations of *M. helenor* also tend to be more dissimilar than sympatric populations of this species, we also tested the convergence of iridescence hypothesis by generating a null distribution of the phenotypic distance only using individuals from the sympatric populations of the Amazonian basin. This method allows for a more conservative estimation of iridescence convergence.

Under the hypothesis of evasive mimicry, the phenotypic variance within population is expected to be lower in sympatric than in allopatric populations of *M. helenor*, as a result of selection favoring resemblance among species. The phenotypic variance within population of *M. helenor* was computed as the trace of the covariance matrix of the PC scores within locality, for males and females separately. We then compared the average values of the trace between sympatric and allopatric populations of *M. helenor* using a *t-*test.

#### Spectral data analysis and statistics using “whole reflectance” dataset

As iridescence refers to any change in reflectance depending on illumination and observation angles, summing up iridescent signals with only three colorimetric variables from the (350–700 nm) range does not capture the whole variation in the visual signal, especially in the UV range. We thus also characterized the iridescence of each individual using the whole spectra obtained at all angular positions, by considering the reflectance at each wavelength as a different variable (400 wavelengths × 13 angular positions). We applied the same protocol as described above on this dataset: we performed a PCA, and retained the first 10 PCs to test for the effect of species, sex, and locality on iridescence using a PERMANOVA. The permutation-based approach to test for the convergence of iridescent signal between sympatric populations was also applied to the distances computed in this 10 PC space depicting the full reflectance variation.

#### Visual modeling

We tested whether the differences in iridescence detected between individuals from different species could be perceived by insectivorous birds using the R package pavo2 2.9.0 (Maia et al., 2019). Indeed, if convergent wing visual signals have been promoted by escape mimicry, predators should generalize the visual signals displayed by individuals from different species in sympatry, likely not perceiving subtle differences in iridescence between them. We specifically used the avian UV-sensitive vision model implemented in pavo (“avg.uv”) as a proxy for predator vision. Among each sympatric locality, the average chromatic (*dS*) and achromatic (*dL*) distances between species and sexes (respectively testing for hue and brightness discrimination) were calculated for each species pair. The error bars show the confidence intervals reflecting the variability of the mean distance under resampling via a bootstrap analysis. When *dS* ≥ 1 and *dL* ≥ 1 just noticeable difference (JND), we considered that the differences in blue could be perceived by an avian predator: this highly sensitive threshold is conservative regarding the discrimination capacities of predators. We also compared predator visual discrimination of predators between *M. helenor* and *M. achilles* in sympatry to allopatric pairs of *M. helenor* to test whether phenotypic convergence between species implies reduced visual discrimination among sympatric individuals from the two sister species (see Supplementary Information 2).

## Results

### Variation in wing iridescence

By estimating the hue, brightness, and chroma from the reflectance spectra obtained at 13 different angles for each individual (Figure 1A), we first compared the iridescent signal displayed in different populations of *M. helenor* and *M. achilles* (see Figure S1 showing the variation of hue measured at different angles of illumination in every sympatric and allopatric locality).

The PCA performed on the hue, brightness, and chroma measured at every angle of illumination then allowed to summarize the combined effects of these colorimetric variables. According to factor loadings, hue variables highly contribute to PC1 and 2 (see Figure 2): angular variation in hue thus appears to be an important driver of the iridescent signal among *Morpho* individuals (Figure S2). The PERMANOVA based on 999 permutations performed on the first 10 PCs shows that iridescence is significantly different between species (*df* = 1, *F* = 27.430, *p-*value = 0.001), sexes (*df* = 1, *F* = 13.718, *p*-value = 0.001), and localities (*df* = 20, *F* = 9.462, *p-*value = 0.001). Additionally, the interactions between species and sex (*df* = 1, *F* = 10.965, *p-*value = 0.001), species and locality (*df* = 9, *F* = 1.944, *p-*value = 0.005), and sex and locality (*df* = 20, *F* = 1.718, *p-*value = 0.001) are also significant. These results highlight the great variation of wing iridescence across Central and South America (see Figure 3A), but also between *Morpho* species. Note that the PCA based on the whole reflectance spectra dataset showed similar results (Figure S3).

**Figure 2.**
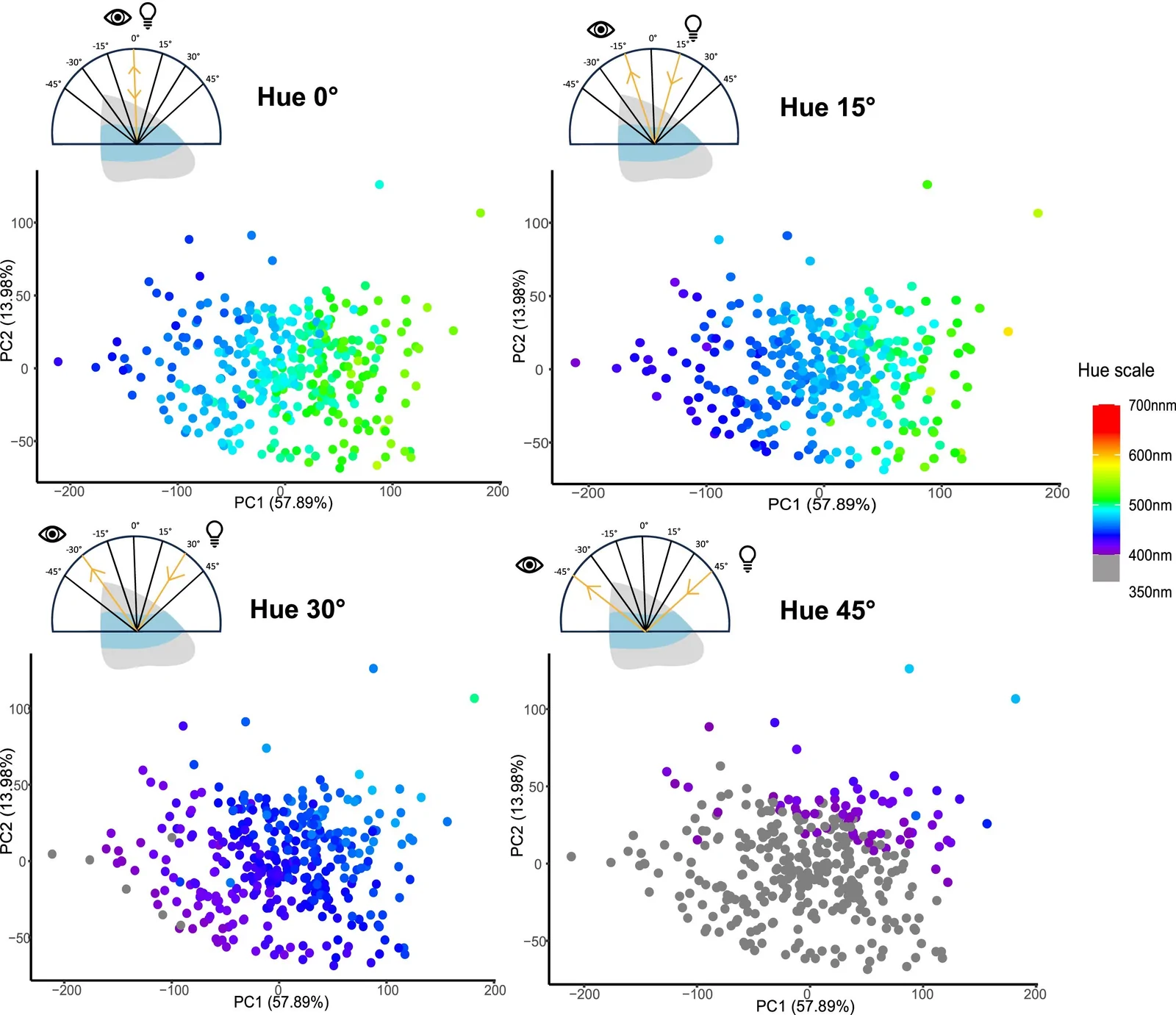
Principal component analysis based on the hue, brightness, and chroma variables measured at every angle of illumination on both wing planes of *Morpho* specimens. Each point on this graph represents the global signal of colorimetric variables depending on the angle of illumination (i.e., iridescence) of a given individual: the closer the points are together, the more similar the iridescence of two individuals is. Note that PC1 and PC2 account for more than 70% of the variance, and that both axes are correlated to hue variables at different angles of illumination (see Figure S2). Here, we used the same PCA but colored each individual dot with their hue value at a 0° angle of illumination, and at 15°, 30°, and 45° on their anterior side of their wings, respectively.

**Figure 3.**
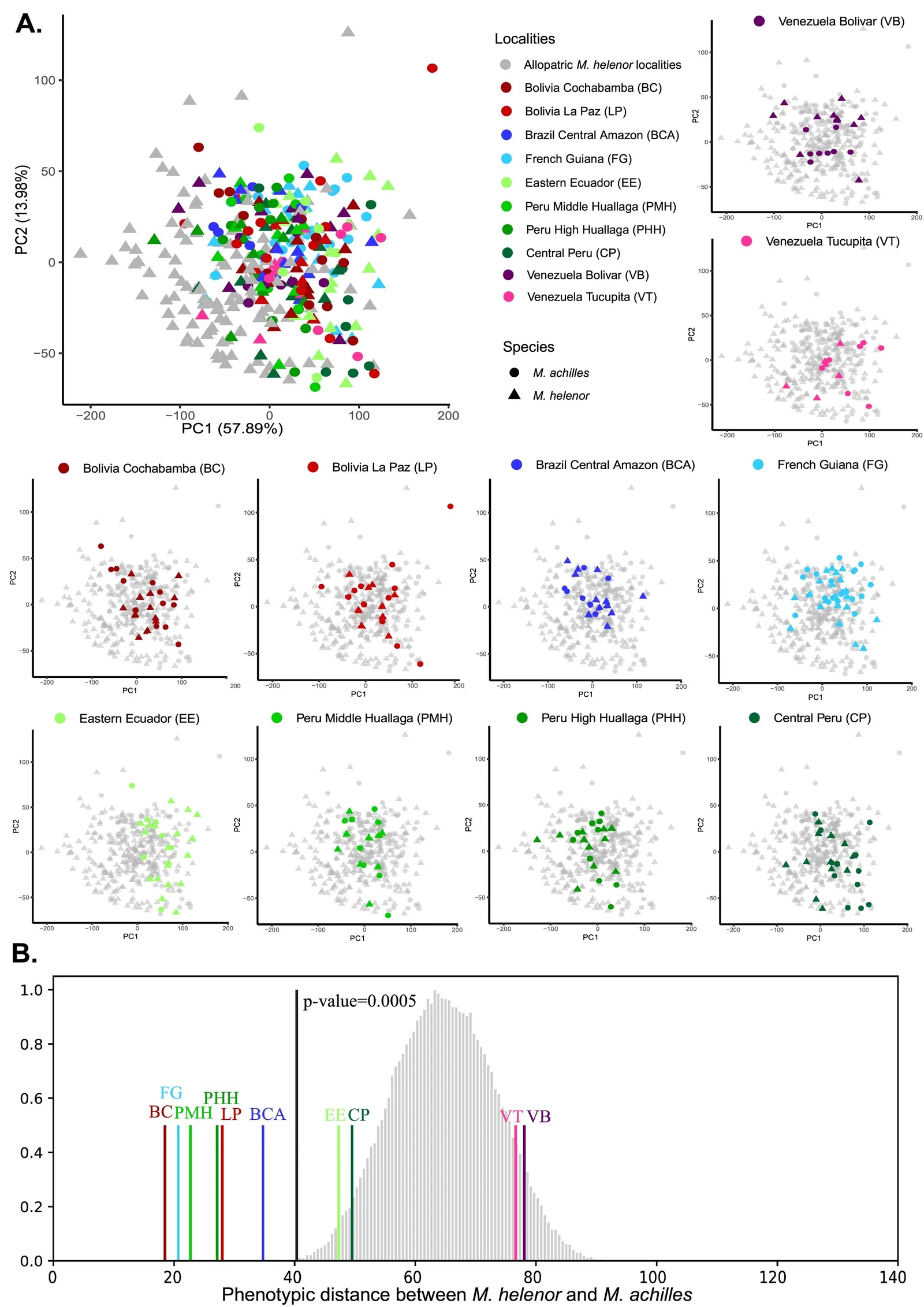
Phenotypic distances between *M. helenor* and *M. achilles* sampled within different geographic regions. (A) Principal component analysis based on the hue, brightness, and chroma variables measured at every angle of illumination on both wing planes of *Morpho* specimens. Each point on this graph represents the global signal of colorimetric variables depending on the angle of illumination (i.e., iridescence) of one individual: the closer the points are together, the more similar the iridescence of two individuals is. Gray points represent the iridescent signal of allopatric *M. helenor* found living in allopatry, while *Morpho* individuals sharing their habitat with closely related species are highlighted in colors according to their locality shown in Figure 1. *Morpho achilles* individuals are represented with circles, *M. helenor* with triangles. Note that PC1 and PC2 account for more than 70% of the variance. (B) Phenotypic distances between *M. helenor* and *M. achilles* within different geographic regions (colored bars), the predicted null phenotypic distribution between species obtained using 10,000 bootstraps, randomly reallocating the different sampling localities among individuals (gray bars), pooling males and females together. The black bar represents the mean phenotypic distance between sympatric species, the *p*-value is based on the number of simulations where the phenotypic distance between species is higher than the mean value of interspecific distance observed in sympatry.

### Signal of convergent iridescence among closely related sympatric *Morpho* species

We used a permutation approach applied on the colorimetric variables to test whether the iridescent signal is more similar among species within sympatric populations than expected at random (Figure 3B). The average phenotypic distance between two heterospecific pairs of individuals was significantly smaller in sympatry than the null distribution simulated by randomizing sympatric and allopatric localities (*p-*value = 0.0005). Except for the two Venezuelan localities (VB and VT), the iridescent coloration in interspecific comparisons was more similar within all sympatric localities. When analyzing the iridescent signals of males and females extracted from the same morphospace separately (Figure S4), similar results were obtained: the mean phenotypic distance between sympatric males (Distmales = 53) is similar to the mean phenotypic distance between females (Distfemales = 54), although differences were observed in some localities. However, the variance of the null distribution of the phenotypic distance of the males (Figure S4A) was lower than the variance of the null distribution of the phenotypic distance of the females (Figure S4B), likely due to a smaller variation of iridescence in males.

Because the allopatric populations of *M. helenor* had divergent phenotypes as compared to the Amazonian ones, we performed the same analysis generating a null distribution using samples from the Amazonian basin only: the signal of phenotypic convergence in sympatry was still found between *M. helenor* and *M. achilles* (Figure S5). This suggests that the contribution of neutral divergence due to isolation by distance or local adaptation, fueling phenotypic divergence between *M. helenor* and *M. achilles* observed in allopatry, is not sufficient to explain the convergence signal detected in sympatry.

The quantification of the variance of iridescence within locality showed that the mean trace of the covariance matrix of sympatric *M. helenor* (*Tsymp* = 3,661, *SD* = 1,018) is lower than the mean trace of the covariance matrix of allopatric *M. helenor* (*Tallo* = 4,613, *SD* = 1,875), suggesting a lower phenotypic variation of iridescence in *M. helenor* populations living in sympatry with *M. achilles*, although the result is not significant (*t*-test: *t* = 1.465, *df* = 15.699, *p-*value = 0.163). The same nonsignificant trend is found for both sexes (*t*-test for males: *t* = 0.729, *df* = 17.561, *p-*value = 0.476; *t*-test for females: *t* = 1.109, *df* = 14.847, *p*-value = 0.2851) but the variance of iridescence is higher in females than in males (males: *Tsymp* = 2,959, *SD* = 800 and *Tallo* = 3,278, *SD* = 1194; females: *Tsymp* = 4,243; SD = 2,075 and *Tallo* = 5,519, *SD* = 2,762).

The results of the permutation tests using the whole reflectance dataset revealed the same tendency (Figure S6), although Venezuelan species appear even more divergent than the rest of the sympatric localities, making the results nonsignificant.

### Discrimination of iridescent wing signals by avian predators

The visual discrimination of the wing iridescence of species in sympatry was estimated using a UV-sensitive avian visual model: we measured the chromatic and achromatic distances between the reflectance spectra obtained at different angles of illumination between *M. helenor*/*M. achilles* individual pairs sampled in the same localities (see Figure S7 for chromatic distances and Figure S8 for achromatic distances). This discrimination dataset is summarized in Table 1. The least convergent *M. helenor*/*M. achilles* pairs distributed in the two Venezuelan localities (VT and VB) are highly discriminated by the modeled visual system of bird observers, especially at the achromatic level. In contrast, most of the significantly convergent populations were not discriminated at most angular positions. However, in some sympatric areas of Peru (PMH and PHH), Bolivia (LP), and Brazil (BCA), bird observers might be able to discriminate slight chromatic and achromatic differences at some specific angles of observations. Nevertheless, the predator visual discrimination of sympatric species is found to be significantly lower than the visual discrimination in allopatric pairs of *M. helenor* (Supplementary Information 2).

**Table 1.** Outline of the visual discrimination analyses of sympatric *Morpho* wings by a UV-sensitive bird created from the results presented in Figures S5 and S6.

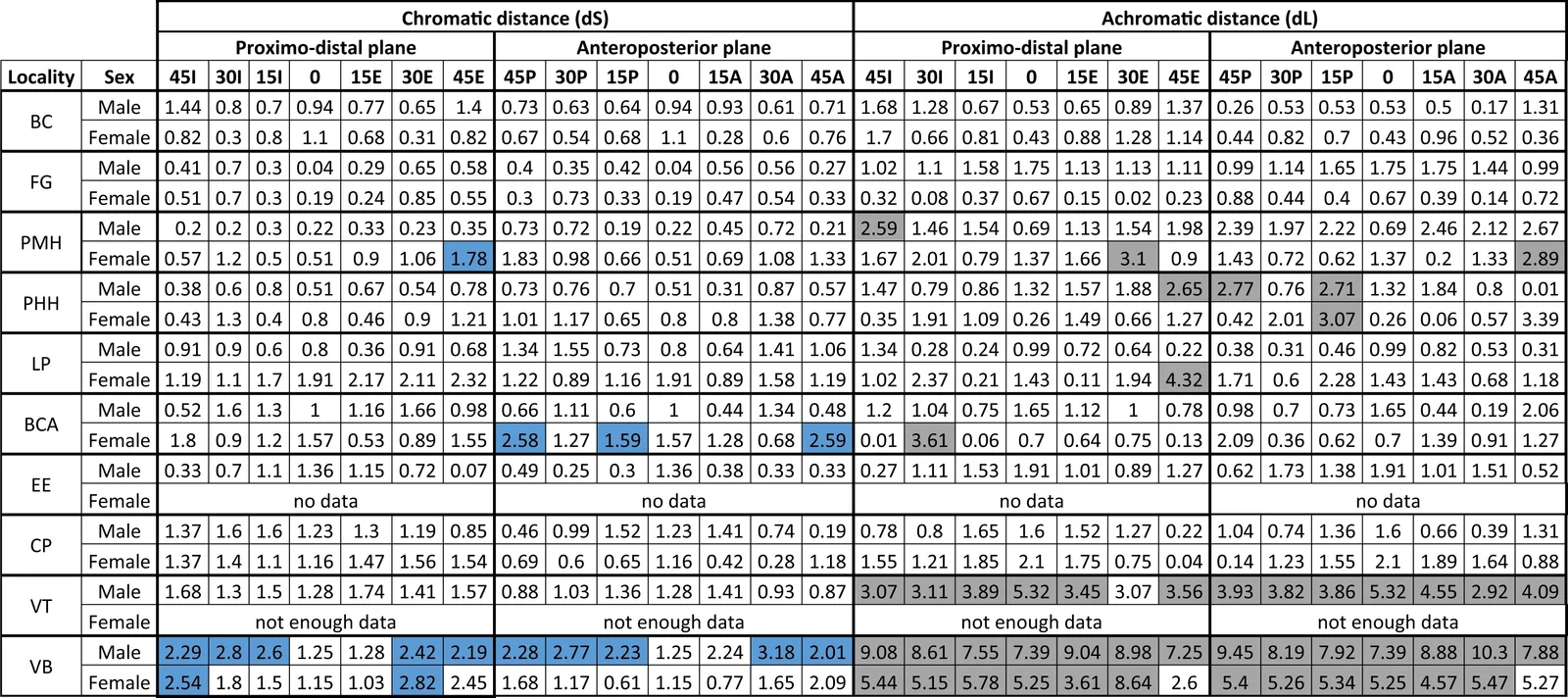

*Note*. The chromatic and achromatic distances of the wing reflectance of sympatric *Morpho* species were measured on the proximodistal and anteroposterior plane of the wings at different angles of illumination, using a UV-sensitive bird visual model, for *M. helenor* and *M. achilles* males and females separately, within each sympatric locality. Their respective value is available in this table. The colored tiles highlight the groups for which the mean chromatic or achromatic distance (including its confidence interval) at a specific angle is higher than 1 just noticeable difference (JND), used as a threshold of visual discrimination. Significant chromatic distances are marked in blue, while significant achromatic distances are marked in gray. Localities are presented from the smaller phenotypic distances (first row) to the larger phenotypic distances (last row).

## Discussion

The comprehensive quantification of the structural coloration of 351 specimens of *Morpho* butterflies allowed to detect a significant signal of repeated convergent evolution of iridescence in multiple geographic areas where the sister species *M. helenor* and *M. achilles* occur in sympatry. This signal is correlated to previously detected local convergences of color pattern in *Morpho* (Llaurens et al., 2021), especially in French Guiana. Our results suggest that indirect survival benefit stemming from predation between species also acts on the evolution of iridescence in this pair of sympatric sister-species

### Convergent iridescence in sympatry: the consequence of local abiotic factors?

The convergent evolution of traits in sympatric species can be due to strong local selective pressures (Yeaman et al., 2016). What could be the main selective forces involved in iridescence convergence in sympatric *Morpho* sister species? First, shared environmental conditions and abiotic factors are expected to promote similar local adaptations between species living in the same habitat (MacLean et al., 2016; Siemers et al., 2024). In sympatry, *M. helenor* and *M. achilles* both live in similar microhabitats in the forest understory, sharing identical light environments and temperatures within each locality (Bouinier et al., 2025; Le Roy et al., 2021). Similar local light conditions could therefore favor the evolution of convergent iridescent patterns. However, the light conditions encountered in the understory are assumed to be rather homogeneous throughout Amazonia, making selection due to variation in light conditions unlikely to explain the variation in iridescence observed among different Amazonian localities. The temperature could also influence the evolution of scale structure and thus the iridescent properties through either a direct effect on the development of scales or an indirect effect due to selection acting on scale evolution. Thermodynamic tests have shown that iridescent butterfly scales may have a higher solar absorbance than pigmentary scales (Bosi et al., 2008), likely due to their light reflecting nanostructures, also involved in color display. However, the precise link between the morphology of scale nanostructures and thermoregulation capacities is not clear: whether subtle nanostructure changes, triggering the reflection of different visual signals (Siddique et al., 2013) could also impact thermoregulation in iridescent blue *Morpho* is currently unknown. Furthermore, heat shock experiments showed that *Morpho* butterflies with iridescent scales have similar thermoregulation capacities compared to non-iridescent *Morpho* butterflies (Bouinier et al., 2025), suggesting that iridescent scales are not necessarily involved in temperature resistance in *Morpho*, and that other factors may impact the evolution of this trait.

### The repeated evolution of convergent iridescent signals is consistent with the evasive mimicry hypothesis

We repeatedly found a high resemblance in iridescence among individuals from *M. helenor* and *M. achilles* within several sympatric localities. Considering that there is no gene flow between these sister species (Le Roy et al., 2021), these phenotypic similarities are unlikely to result from repeated events of introgression, as observed in other mimetic butterfly species living in sympatry (Dasmahapatra et al., 2012). These similarities could rather stem from shared ancestral polymorphism or independent evolution of similar phenotypic variations in these two species. In either case, the observed phenotypic convergence would be favored by the behaviors of the local communities of predators, although direct evidences from behavioral experiments with wild birds remain scarce (but see Pinheiro et al. 2016 ). *Morpho* iridescence is a visual cue that could promote the escape of these butterflies from predators (Pinheiro, 1996; Vieira-Silva et al., 2024; Young, 1971) and facilitate predator learning via their conspicuousness (Pinheiro & Campos, 2019): these factors could have promoted the observed convergent evolution of iridescent signals via escape mimicry, especially of the hue variation of their wings.

Interestingly, the variance of the iridescent signal was higher among females than among males. Because *Morpho* males typically patrol in open, exposed habitats where they are more visible to predators, whereas females usually remain concealed in the forest understory (Le Roy et al., 2021), males are likely subject to stronger predation pressures while other forces such as sexual selection might constrain the evolution of female wings. This may select for greater uniformity in the iridescent signal of males, leading to reduced phenotypic variance compared to females.

Although we found a repeated evolution of convergent iridescent colors in sympatry, the mean iridescent signal varied between localities. This geographic heterogeneity of iridescent signals may be driven by local variation in predator communities, as found in *Heliconius* butterflies: variations in local predator communities are indeed identified as driving the emergence of different mimicry rings between *H. melpomene* and *H. erato* for example (Mallet, 2010; Mallet & Gilbert, 1995). Models show that predator perception, preferences, and behaviors may promote the selection of diverse mimetic patterns in prey from different localities maintained by positive frequency-dependent selection (Aubier & Sherratt, 2015), as shown in butterflies but also in frogs (Symula et al., 2001), bees and wasps (Chatelain et al., 2023), or ants (Wilson et al., 2012).

By estimating the visual discrimination of *Morpho* iridescence using a vision model corresponding to UV-sensitive birds, we also showed that the minute differences observed in sympatric areas between individuals from different species are unlikely to be detected by birds in most geographic areas, unlike in allopatric populations. Iridescent wing coloration exhibited by the two sympatric species of *Morpho* is thus perceived as visually highly similar by birds, consistent with the hypothesis of evasive mimicry.

### Direct and indirect effect of iridescence on predator attacks

Differences between species in reflectance displayed at certain angles are nevertheless observed in some localities where convergence in iridescence was detected, highlighting that the local convergence can be somewhat limited. Such limitation could be due to divergent developmental constraints in the two species or differences in the available standing genetic variations causing iridescence evolution in the two species. Furthermore, even if detected by birds, minute differences in reflectance at certain angles might not prevent bird generalization and avoidance, as shown by the prevalence of imperfect mimicry among aposematic prey (Kikuchi & Pfennig, 2013). For instance, predators tend to generalize aposematic signals and be less sensitive to slight phenotypic variation of aposematic prey in communities exhibiting more diverse visual signals (Ihalainen et al., 2012), allowing the evolution of nonaccurate mimicry. The iridescent blue color of *Morpho* being relatively rare within the highly diverse prey community they live in, the minute differences in iridescence between species, even if perceived by predators, may not be significant enough to prevent generalization.

Furthermore, in *Morpho* butterflies, iridescence directly participates in predator escape due to the confusion effect limiting the catching success in case of predator attack. Selection for accurate mimicry between iridescent evasive species might be less stringent than for aposematic prey displaying pigmentary colors where color itself has no effect on survival during the attack. Such direct survival advantage provided by iridescence during predator attack may thus limit the survival advantage brought by mimicry and weaken the selection on resemblance. Identifying what part of the iridescent signal generates confusion and is memorized by predators is now needed to assess how predator behavior affects the evolution of iridescent wing pattern in *Morpho*. In *Morpho*, the relative importance of flashes during flight or color variation as evasive/dissuasive signals for predators has not been studied. For instance, dynamic colors might reduce predation success when a prey is moving (Murali, 2018), as well as flashing signals involving rapid changes of brightness (Loeffler-Henry et al., 2021): what could be the incidence of these effects combined in *Morpho* butterflies? Whether all the colors displayed on the iridescent spectrum of *Morpho* butterflies are important in predator confusion and learning is still an open question.

Interestingly, no signal of phenotypic convergence was found between the sympatric *M. helenor* and *M. achilles* sister species in two localities in Venezuela (VT and VB). This result is consistent with those of Llaurens et al. (2021), showing that the width of their blue band also diverges in these populations. This result could be due to our delimitation of populations, pooling together individuals from different locations, actually submitted to different predator communities. Venezuela is also a contact zone between Amazonian and Central American populations of *M. helenor*. Within *M. helenor*, gene flow between these two areas is likely very low because the Andes mountains are an important geographic barrier. Subspecies of *M. helenor* living in these separated areas indeed display very dissimilar iridescent colors and patterns (Blandin & Purser, 2013). The gene flow of *M. helenor* populations from central America to the Venezuelan populations likely generates greater phenotypic variability in *M. helenor* as compared to *M. achilles* whose populations are strictly Amazonian, resulting in more divergent iridescent signals in this area.

## Conclusion

Our results are consistent with a primary effect of selection exerted by local predator communities on the evolution of convergent iridescent colorations between closely related *M. helenor* and *M. achilles* living in sympatry. Behavioral experiments investigating the effect of variation in iridescence pattern on predators are now needed to validate this hypothesis.

## Supplementary Material

qrag028_Supplemental_File

## Data Availability

The data and scripts used in this article are available in a Zenodo repository: https://doi.org/10.5281/zenodo.18600542
