# Supplementary material for: Repeated evolution of convergent iridescence in closely related species of Morpho butterflies living in sympatry
Source: Evol Lett. 2026 Jul 10;10(5):493–504. doi: 10.1093/evlett/qrag028 (PMC13629238; doi:10.1093/evlett/qrag028)
Supplement: Supplementary file 1 [file qrag028_supplemental_file.docx]

**Supplementary information: Repeated evolution of convergent iridescence in closely-related species of *Morpho* butterflies living in sympatry**

Joséphine Ledamoisel^1,2^, Vincent Debat^1,2^, Violaine Llaurens^1^

^1^ Centre Interdisciplinaire de Recherche en Biologie (UMR 7241, Collège de France/CNRS/INSERM), Collège de France 11 place Marcelin Berthelot 75005 Paris, France

^2^ Institut de Systématique, Evolution et Biodiversité (ISYEB UMR 7205 CNRS/MNHN/SU/EPHE/UA), Muséum National d’Histoire Naturelle - CP50, 45 rue Buffon, 75005 PARIS, France

Corresponding author: Joséphine Ledamoisel (josephine.ledamoisel@outlook.fr)

**Supplementary Information**

**Table S1:** Number of specimens used for the wing reflectance measurements from the collection of the National Museum of Natural History (Paris, France). The subspecies name among each geographical locality is given according to Blandin (2007), sympatric localities are highlighted in black and allopatric localities are highlighted in red.

| **Biogeo-graphic region** | **Species co-occurrence** | **Sampling zone** | **Code** | ***M. helenor*** | | | ***M. achilles*** | | |  |
| --- | --- | --- | --- | --- | --- | --- | --- | --- | --- | --- |
|  |  |  |  |  |  |  |  |  |  |  |
|  |  |  |  | **Subspecies** | ♂ | ♀ | Subspecies | ♂ | ♀ | Total |
| *trans-andean* | **Allopatry** | **Central America** | **CA** | *montezuma* | 6 | 6 | - | - | - | 12 |
|  | **Allopatry** | **Costa Rica** | **CR** | *carillensis* | 6 | 6 |  |  |  | 12 |
|  | **Allopatry** | **Costa Rica/Panama** | **CRP** | *limpida* | 6 | 1 | - | - | - | 7 |
|  | **Allopatry** | **Western Colombia** | **WP** | *microphthalmus* | 6 | 6 | - | - | - | 12 |
|  | **Allopatry** | **Central Colombia** | **CC** | *peleides* | 6 | 1 | - | - | - | 7 |
| *cis*-Andean | **Sympatry** | **Eastern Ecuador** | **EE** | *theodorus* | 10 | 10 | *phokylides* | 6 | 0 | 26 |
|  | **Allopatry** | **Western Ecuador** | **WE** | *bristowi* | 10 | 10 | - | - | - | 20 |
|  | **Allopatry** | **Peru Middle Marañon** | **PM** | *charapensis* | 6 | 6 | - | - | - | 12 |
|  | **Sympatry** | **Peru** **Middle Huallaga** | **PMH** | *theodorus* | 6 | 3 | *phokylides* | 6 | 2 | 17 |
|  | **Sympatry** | **Peru** **High Huallaga** | **PHH** | *lacommei* | 6 | 5 | *fagardi* | 6 | 4 | 21 |
|  | **Sympatry** | **Central Peru** | **CP** | *papirius* | 6 | 6 | *agamedes* | 6 | 6 | 24 |
|  | **Sympatry** | **Bolivia La Paz** | **LP** | *coelestis* | 6 | 4 | *songo* | 6 | 6 | 22 |
|  | **Sympatry** | **Bolivia Cochabamba** | **BC** | *coelestis* | 6 | 6 | *vitrea* | 6 | 6 | 24 |
|  | **Sympatry** | **Brazil Central Amazon** | **BCA** | *helenor* | 6 | 6 | *achilles* | 6 | 1 | 19 |
|  | **Sympatry** | **Venezuela Bolívar** | **VB** | *tepuina* | 6 | 5 | *glaisi* | 6 | 2 | 19 |
|  | **Sympatry** | **Venezuela Tucupita** | **VT** | *tucupita* | 5 | 1 | *guaraunos* | 6 | 1 | 13 |
|  | **Allopatry** | **Venezuela** **El Pao** | **EP** | *ululina* | 6 | 3 | *-* |  | - | 9 |
|  | **Sympatry** | **Surinam/French Guiana/Northern Pará** | **SFR** | *helenor* | 10 | 10 | *achilles* | 10 | 10 | 40 |
| Atlantic | **Allopatry** | **Argentina Misiones** | **AM** | *achillides* | 6 | 5 | - | - | - | 11 |
|  | **Allopatry** | **Brazil** **Rio de Janeiro** | **BR** | *achillaena* | 6 | 6 | - | - | - | 12 |
|  | **Allopatry** | **Brazil** **João Pessoa** | **BJ** | *anakreon* | 6 | 6 | - | - | - | 12 |
| Total |  | | | | 137 | 112 |  | 64 | 38 | **351** |

**A.**

**B.**

**Figure S1:** Hue iridescence measured among *M. helenor*, *M. achilles* and *M. granadensis* in (A) sympatric and (B) allopatric populations. Each box represents a studied locality, and contains the picture of male (top) and female (bottom) wing morphology belonging to sympatric sister species (among sympatric locations) or to *M. helenor* only (among allopatric locations). For each sex, the mean Hue value among individuals was calculated using the reflectance spectra measured at every measured studied angle, either on the Antero-posterior (first row) or on the Proximo-distal (second row) plane of the wings. Mean hues ranging from 400nm to 700nm are represented using a RGB colour scale ranging from purple to red following the colour variations observed on the visible light spectrum. Mean hues ranging from 350nm to 400nm in the UV-range are coloured in grey. Note that this colour representation is only illustrative and do not simulate butterflies’ nor predators’ vision.

**Table S2:** Repeatability (R) of the measurements of iridescence performed on the wings of *M. helenor* and *M. achilles*. The repeatability was measured over 3 repeated measurements of Brightness, Hue, and Chroma at every angle of illumination for n=10 male individuals

| **R** | **SE** | **Emp.2.5%** | **Emp.97.5%** | **P-value** | **Variable** | **Angle_ID** |
| --- | --- | --- | --- | --- | --- | --- |
| 0.9067906 | 0.10722253 | 0.54062614 | 0.9678824 | 8.66E-14 | Chroma | _0_ |
| 0.8847553 | 0.12717482 | 0.50960517 | 0.95997 | 5.51E-11 | Brightness | _0_ |
| 0.9339046 | 0.08466289 | 0.70715867 | 0.9781464 | 5.06E-16 | Hue | _0_ |
| 0.9424534 | 0.07287288 | 0.75157849 | 0.9818729 | 7.37E-16 | Chroma | _15A_ |
| 0.9028747 | 0.10919185 | 0.5525725 | 0.9609625 | 1.69E-10 | Brightness | _15A_ |
| 0.8829328 | 0.13664136 | 0.47560276 | 0.9626787 | 3.10E-10 | Hue | _15A_ |
| 0.6540093 | 0.20224753 | 0.06329693 | 0.8675049 | 1.07E-06 | Chroma | _30A_ |
| 0.6513324 | 0.19840671 | 0.05006459 | 0.857428 | 5.25E-05 | Brightness | _30A_ |
| 0.9495709 | 0.06207607 | 0.73267626 | 0.9855204 | 8.23E-14 | Hue | _30A_ |
| 0.8771626 | 0.13119122 | 0.46294819 | 0.9582205 | 1.51E-13 | Chroma | _45A_ |
| 0.5928395 | 0.21136452 | 0.03105701 | 0.8352327 | 1.36E-03 | Brightness | _45A_ |
| 0.9249303 | 0.08475622 | 0.65955664 | 0.9735729 | 1.63E-12 | Hue | _45A_ |
| 0.898254 | 0.10380468 | 0.57610384 | 0.961901 | 3.14E-13 | Chroma | _15E_ |
| 0.8170788 | 0.16335849 | 0.30449012 | 0.9358691 | 4.44E-09 | Brightness | _15E_ |
| 0.9206384 | 0.10425039 | 0.57250576 | 0.9715503 | 9.92E-14 | Hue | _15E_ |
| 0.437042 | 0.19698907 | 0 | 0.7374238 | 2.45E-03 | Chroma | _30E_ |
| 0.7324226 | 0.19088852 | 0.17148289 | 0.9079994 | 4.81E-07 | Brightness | _30E_ |
| 0.8344855 | 0.14128865 | 0.3636496 | 0.9439499 | 6.67E-10 | Hue | _30E_ |
| 0.5111851 | 0.22316633 | 0 | 0.7959772 | 1.58E-02 | Chroma | _45E_ |
| 0.5572016 | 0.21768389 | 0 | 0.8189653 | 3.21E-03 | Brightness | _45E_ |
| 0.7727012 | 0.17461998 | 0.21634691 | 0.9152582 | 9.54E-09 | Hue | _45E_ |
| 0.8685001 | 0.12823047 | 0.45825354 | 0.9525691 | 2.44E-11 | Chroma | _15I_ |
| 0.7730533 | 0.16979886 | 0.28211422 | 0.91694 | 2.92E-08 | Brightness | _15I_ |
| 0.906945 | 0.09900359 | 0.61486806 | 0.969312 | 3.66E-13 | Hue | _15I_ |
| 0.8318865 | 0.14229666 | 0.38625546 | 0.9398735 | 1.88E-11 | Chroma | _30I_ |
| 0.7695286 | 0.17562081 | 0.21886196 | 0.912323 | 7.38E-09 | Brightness | _30I_ |
| 0.8515021 | 0.12345096 | 0.52304858 | 0.9485898 | 2.12E-10 | Hue | _30I_ |
| 0.5569942 | 0.22607385 | 0 | 0.8164967 | 1.56E-03 | Chroma | _45I_ |
| 0.4566122 | 0.21863097 | 0 | 0.7498699 | 6.76E-03 | Brightness | _45I_ |
| 0.8171747 | 0.17287445 | 0.24048107 | 0.9320763 | 5.80E-09 | Hue | _45I_ |
| 0.8957745 | 0.12597941 | 0.48261534 | 0.966791 | 4.45E-12 | Chroma | _15P_ |
| 0.8487842 | 0.14108403 | 0.39921805 | 0.946995 | 7.21E-09 | Brightness | _15P_ |
| 0.8492725 | 0.1382803 | 0.4080627 | 0.9469374 | 9.85E-08 | Hue | _15P_ |
| 0.7263971 | 0.19584676 | 0.09132553 | 0.8919757 | 2.22E-06 | Chroma | _30P_ |
| 0.7612096 | 0.18553053 | 0.1984362 | 0.9053677 | 1.18E-05 | Brightness | _30P_ |
| 0.9592962 | 0.06288081 | 0.79029857 | 0.9867222 | 3.06E-15 | Hue | _30P_ |
| 0.8787944 | 0.12662752 | 0.46976708 | 0.9612275 | 9.14E-12 | Chroma | _45P_ |
| 0.8633325 | 0.12730209 | 0.4749708 | 0.9524398 | 7.28E-09 | Brightness | _45P_ |
| 0.9408253 | 0.06504858 | 0.72597826 | 0.9820759 | 2.80E-15 | Hue | _45P_ |

**Supplementary information 1: Analysis of the impact of the sampling date on the variation of iridescence measured in a subset of studied individuals**

As a structural colour, blue iridescence is produced by the reflection of light on chitin nanostructures found on Morpho wing scales. Hence the only source of degradation over time would be a physical alteration of the wings. However, since chitin is an extremely resistant material (Vincent & Wegst, 2004), wear due to aging is expected to be minimal. As melanin pigments also contribute to *Morpho* blue coloration, a decay of these pigments due to long light exposure might alter reflectance. However, such an effect would be restricted to overall hue and should not affect angular dependence. In addition, we only included almost perfectly preserved specimens conserved in dark and dry conditions, with intact wing surface.

However, because sampling dates and location are partially correlated, we conducted statistical analyses to test whether accounting for the sampling date might alter the results of our study. We only have certainty on the sampling dates of *n* = 258 specimens out of the 351 specimens mentioned in our study, and thus used this sub-sample to test for the effect of sampling date on iridescence convergence.

First, we run the PERMANOVA based on 999 permutations performed on the first 10 PCs of the PCA based on the description of **iridescence based on Brightness, Hue, and Chroma** (the main model presented in the result section) on the *n* = 258 dated individuals and including the sampling date of the specimen as a model parameter (Table SI1.1). The model clearly shows no significant effect of “Date”.

*Table SI1.1: Results of the PERMANOVA performed on the first 10 PC of the PCA based on the description of iridescence based on Brightness, Hue and Chroma*

|  | Df | SumOfSqs | R2 | F | Pr(>F) |
| --- | --- | --- | --- | --- | --- |
| Species | 1 | 40401.746 | 0.02416886 | 9.7908096 | 0.001 |
| Sex | 1 | 36973.203 | 0.02211786 | 8.9599492 | 0.002 |
| Locality | 19 | 537615.492 | 0.32160871 | 6.8570395 | 0.001 |
| Date | 1 | 7877.293 | 0.0047123 | 1.9089541 | 0.123 |
| Species*Sex | 1 | 29693.842 | 0.01776325 | 7.1958959 | 0.003 |
| Species*Locality | 6 | 36272.49 | 0.02169868 | 1.4650235 | 0.117 |
| Sex*Locality | 18 | 117468.054 | 0.07027095 | 1.5814874 | 0.014 |
| Species*Sex*Locality | 4 | 15284.234 | 0.00914323 | 0.9259812 | 0.493 |
| Residual | 206 | 850058.362 | 0.50851617 | NA | NA |
| Total | 257 | 1671644.72 | 1 | NA | NA |

We have specimens collected at different time within some localities, and different localities sampled at the same periods allowing to disentangle the effect of localities from the effect of the sampling date. We thus computed the p-values associated with the effect of Sex, Species and Date parameters obtained by performing similar PERMANOVAs in each separated locality, when controlling for the sampling dates (Table SI1.2). This table allows to capture the status of the studied locality (Interaction), the sampling date range found among each locality (Date_Range), and the number of individuals for which “date” data is available compared to the total number of individuals with iridescence measurements (Sampling_Size).

*Table SI1.2: Locality-dependent variations of sampling dates and their statistical effect on iridescence variation (PERMANOVAs)*

| **Region** | **Interactions** | **Date_Range** | **Sample_Size** | **pval_Date** | **pval_Sexe** | **pval_Species** | **pval-Sex*Species** |
| --- | --- | --- | --- | --- | --- | --- | --- |
| Argentina_Misiones | Allopatry | 1955-1994 | n=4/11 | 0.4166667 | 0.25 | _ | _ |
| Brazil_Joao | Allopatry | 1974_1986 | n=9/12 | 0.543 | 0.083 | _ | _ |
| Brazil_Rio | Allopatry | 1860-1961 | n=12/12 | 0.254 | 0.339 | _ | _ |
| Central_Columbia | Allopatry | 1862_1920 | n=7/7 | 0.68 | 0.452 | _ | _ |
| Costa_Rica | Allopatry | 1980_1983 | n=7/12 | 0.582 | 0.302 | _ | _ |
| Costa_Rica_Panam | Allopatry | 1978-1989 | n=7/7 | 0.747 | 0.262 | _ | _ |
| Mexico | Allopatry | 1877-1976 | n=9/12 | 0.703 | 0.01 | _ | _ |
| Peru_Middle_Maranon | Allopatry | 1971-2014 | n=12/12 | 0.601 | 0.194 | _ | _ |
| Venezuela_El_Pao | Allopatry | 1961-1975 | n=9/9 | 0.749 | 0.134 | _ | _ |
| West_Columbia | Allopatry | 1967-1975 | n=8/12 | 0.785 | 0.513 | _ | _ |
| Western_Ecuador | Allopatry | 2023 | n=20/20 | only 1 sampling date | | | |
| Bolivia_Cochabamba | Sympatry | 2008 | n=6/24 | only 1 sampling date | | | |
| Brazil_Central_Amazon | Sympatry | 1973-1986 | n=19/19 | 0.661 | 0.46 | 0.202 | 0.706 |
| Central_Peru | Sympatry | 1973-1987 | n=12/24 | 0.737 | 0.431 | 0.439 | 0.739 |
| Eastern_Ecuador | Sympatry | 1970-2023 | n=25/25 | 0.797 | 0.101 | 0.059 | _ |
| French_Guiana | Sympatry | 2023 | n=40 | only 1 sampling date | | | |
| La_Paz_Bolivia | Sympatry | 1903-2020 | n=12/22 | 0.595 | 0.034 | 0.929 | _ |
| Peru_High_Huallaga | Sympatry | 1958-1960 | n=3/20 | not enough individuals | | | |
| Peru_Middle_Huallaga | Sympatry | 2005-2018 | n=17/17 | **0.041*** | 0.011 | 0.443 | 0.299 |
| Venezuela_Bolivar | Sympatry | 1986-2005 | n=18/19 | 0.759 | 0.807 | 0.001 | 0.048 |
| Venezuela_Tucupita | Sympatry | no date available | | | | | |

Among all studied populations, the “Date” parameter is found to have a significant effect on the observed variation of iridescence in only 1 locality (“Peru_Middle_Huallaga”), despite not being the locality with the largest Date_Range. We conclude that the sampling date of our specimens has no effect on the observed variation of iridescence among localities. The observed differences between sympatry and allopatry are thus very likely a genuine ecological effect rather than a bias due to collecting dates.

**Supplementary information 2: Comparison of the predator visual discrimination of sympatric *M. helenor* and *M. achilles* to the predator visual discrimination of pairs of allopatric *M. helenor***

If the chromatic and achromatic values in allopatry are already low (typically below 1 JND), then smaller discrimination values in sympatry cannot be due to predation selection. To test for this, we thus performed additional visual modelling analyses by measuring the chromatic distance (dS) and achromatic distance (dL) between *M. helenor* individuals sampled in 5 pairs of allopatric populations: Central America (CA) vs. Costa Rica (CR), Costa Rica (CR) vs. Panama (CRP), Western Colombia (WC) vs. Central Colombia (CC), Western Ecuador (WE) vs. Peru (PM), Argentina (AM) vs. Brazil (BR). We specifically selected these pairs of allopatric populations because they are geographically close to each other, which minimises the effects of large-scale geographic variation and enables a more direct comparison with sympatric populations.

*Table SI2.1: The chromatic and achromatic distances of the wing reflectance of allopatric M. helenor were measured on the proximo-distal and antero-posterior plane of the wings at different angles of illumination, using a UV-sensitive bird visual model, for males and females separately. The coloured tiles highlight the groups for which the mean chromatic or achromatic distance (including its confidence interval) at a specific angle is higher than 1 Just Noticeable Difference (JND), used as a threshold of visual discrimination.*

These results show that the blue iridescence of different *M. helenor* individuals sampled from two allopatric populations are more likely discriminated by putative predators than *M. helenor* and *M. achilles* individuals living in sympatry (*Table SI2.1*). We used a linear model to test for the effect of the population interaction type (sympatry and allopatry), the angle of measured iridescence and sex on the variation of dS (*Table SI2.2*) and dL (*Table SI2.3*).

*Table SI2.2: Results of the linear model testing for the effect of the population interaction type (Pop interaction), the angle of measurement (Angle) and the Sex (Sex) on the variation of chromatic distances (dS).*

|  | Df | Sum.Sq | Mean.Sq | F.value | p-value |
| --- | --- | --- | --- | --- | --- |
| Pop interaction | 1 | 102.930334 | 102.930334 | 200.864219 | 3.30E-35 |
| Angle | 12 | 13.416314 | 1.1180262 | 2.181781 | 1.26E-02 |
| Sex | 1 | 6.441209 | 6.4412088 | 12.569748 | 4.55E-04 |
| Pop interaction:Angle | 12 | 11.540144 | 0.9616786 | 1.876675 | 3.67E-02 |
| Residuals | 298 | 152.706339 | 0.5124374 | NA | NA |

*Table SI2.3: Results of the linear model testing for the effect of the population interaction type (Pop interaction), the angle of measurement (Angle) and the Sex (Sex) on the variation of achromatic distances (dL).*

|  | Df | Sum.Sq | Mean.Sq | F.value | p-value |
| --- | --- | --- | --- | --- | --- |
| Pop interaction | 1 | 91.2484 | 91.248399 | 42.925481 | 2.49E-10 |
| Angle | 12 | 14.57171 | 1.214309 | 0.5712407 | 8.65E-01 |
| Sex | 1 | 17.58438 | 17.584384 | 8.272125 | 4.32E-03 |
| Pop interaction:Angle | 12 | 14.19143 | 1.18262 | 0.5563332 | 8.76E-01 |
| Residuals | 298 | 633.47043 | 2.12574 | NA | NA |

*Table SI2.2* results show that there is a clear difference of dS between sympatric and allopatric populations (df=1, F=200.86, p-value=3.30E-35), between angles (df=12, F=2.18, p-value=0.013) and between sexes (df=1, F=12.57, p-value=4.55E-04). The fact that the interaction between “Pop interaction” and “Angle” is significant (df=12, F=1.88, p-value=3.67E-02) reveals that the amount of divergence found between sympatric and allopatric populations depends on the studied angle. *Table SI2.3* results show that there is a difference of dL between sympatric and allopatric populations (df=1, F=42.93, p-value=2.49E-10) and between sexes (df=1, F=8.27, p-value=4.32E-03) but not between angles.

Overall, these results suggest that chromatic and achromatic distances are higher between allopatric *M. helenor* individuals compared to sympatric *M. helenor* and *M. achilles* individuals, with a stronger difference found among chromatic distances compared to achromatic distances. Different species living in sympatry tend to be less recognisable than the same species living in different habitats, which is consistent with a putative impact of predator perception on the evolution of convergent colour patterns.

**Figure S2:** Visual representation of the contribution to the PCA representation of Hue, Brightness and Chroma variables measured on the Proximo-distal and Antero-posterior plane at different angles of illumination: both axes are correlated to Hue variables at different angles of illumination.

**A.**

**B.**

| **Variables** | **Df** | **SumOfSqs** | **R2** | **F** | **Pr(>F)** |
| --- | --- | --- | --- | --- | --- |
| Species | 1 | 7121217 | 0.04256886 | 27.138524 | **0.001**** |
| Sex | 1 | 9215814 | 0.05508984 | 35.120908 | **0.001**** |
| Locality | 20 | 45799237 | 0.27377643 | 8.726906 | **0.001**** |
| Species*Sex | 1 | 1782905 | 0.01065776 | 6.794544 | **0.002**** |
| Species*Locality | 9 | 13959759 | 0.08344796 | 5.911088 | **0.001**** |
| Sex*Locality | 20 | 10905046 | 0.06518765 | 2.077923 | **0.001**** |
| Species*Sex*Locality | 8 | 2406292 | 0.01438421 | 1.146279 | 0.281 |
| Residual | 290 | 76096725 | 0.45488727 | NA | NA |
| Total | 350 | 167286996 | 1 | NA | NA |

**Figure S3**: (A) Principal component analysis based on the “whole reflectance” dataset, using the reflectance of every nanometre for each angle of illumination as a variable, accounting for the variation of colouration of *Morpho* butterflies on both wing planes. Each point on this graph represents the global iridescence signal of 1 individual, while considering angle variation: the closer the points are together, the more similar the iridescence of two individuals is. Grey points represent the iridescent signal of allopatric *M. helenor* found living in allopatry, while *Morpho* individuals sharing their habitat with closely-related species are highlighted in colours according to their locality shown on Figure 1. *M. achilles* individuals are represented with circles, and *M. helenor* with triangles. Note that PC1 and PC2 account for more than 60% of the variance. (B) PERMANOVA results testing for the effect of Species, Sex, Locality and the interactions between those variables on the first 10 PCs of the “whole reflectance” PCA. Similar to the PERMANOVA performed on the colorimetric variable coordinates in the main text, this method allows to detect a significant effect of species, sex, locality, and of the interactions between those terms, suggesting that iridescence is highly diversified, both geographically and between species and subspecies.

**Figure S4**: Phenotypic distances measured using the coordinates based on the Brightness-Hue-Chroma dataset, between *M. helenor* and *M. achilles* sharing the same locality (coloured bars) and the predicted null phenotypic distribution between species obtained using 10,000 bootstraps, randomly reallocating the different sampling zone between sympatric and allopatric species (grey bars). Graph (B) presents the global phenotypic distances measured by pooling males together, while graph (B) presents the global phenotypic distances measured by pooling females together. The black bar represents the mean phenotypic distance between sympatric species, the p-value is based in the number of simulations where the phenotypic distance between species is higher than the mean value of inter-specific distance observed in sympatry. Note that even if the mean phenotypic distance of males and females is similar, the phenotypic distance measured in some localities (such as LP, BC, CP for example) can differ between sexes.

**Figure S5:** Phenotypic distances between M. helenor and M. achilles measured using the Brightness-Hue-Chroma dataset but only keeping the populations found in the Amazonian Basin (10 sympatric locations: FG, PMH, LP, BCA, PHH, EE, CP, BC, VT, VB; 2 allopatric locations where M. helenor is found isolated: PM and EP). The predicted null phenotypic distribution between species obtained using 10,000 bootstraps, randomly reallocating the different sampling zone between sympatric and allopatric species are shown in grey, and the phenotypic distance of each sympatric locations are shown in colours. Graph (A) present the global phenotypic distances measured by pooling males and females together, while graphs (B) and (C) only display the phenotypic distances measured between males and females only respectively. The black bar represents the mean phenotypic distance between sympatric species, the p-value is based in the number of simulations where the phenotypic distance between species is higher than the mean value of inter-specific distance observed in sympatry.

**Figure S6:** Phenotypic distances measured using the “whole reflectance” coordinates, between *M. helenor* and *M. achilles* sharing the same locality (coloured bars) and the predicted null phenotypic distribution between species obtained using 10,000 bootstraps, randomly reallocating the different sampling zone between sympatric and allopatric species (grey bars). Graph (A) present the global phenotypic distances measured by pooling males and females together, while graphs (B) and (C) only display the phenotypic distances measured between males and females only respectively. The black bar represents the mean phenotypic distance between sympatric species, the p-value is based in the number of simulations where the phenotypic distance between species is higher than the mean value of inter-specific distance observed in sympatry. Compared to the permutation results obtained with the colorimetric variables dataset, this method detects a greater divergence of *M. helenor* and *M. achilles* iridescent colour patterns in Venezuela, strongly influencing the mean phenotypic distance between sympatric species (black bar). However, a convergence of iridescent signal is still detected in the other sympatric populations. Furthermore, phenotypic resemblance is higher in sympatric females than in sympatric males.

**Figure S7:** Chromatic distances (i.e. the visual discrimination rate of hue by an avian visual model) of the wing reflectance measured (A) on the Antero-posterior and (B) the Proximo-distal planes of Morpho wings. The chromatic distances have been measured separately between females (first row of each figure) and males (second row of each figure) of the two species sharing each sympatric location, in order to account for sexual dimorphism. Among each locality, the tested angles are displayed from right to left following the order: [-45°, -30°, -15°, 0°, 15°, 30°, 45°] on the Antero-posterior plane, and [-45°I, -30°I, -15°I, 0°, 15°, 30°, 45°] on the Proximo-distal plane. The dotted line represents the threshold of discrimination of JND=1: visual discrimination is considered possible if the measured the mean chromatic distance (coloured circle) and its confidence interval (coloured vertical line) are superior to the threshold.

**Figure S8:** Achromatic distances (i.e. the visual discrimination rate of brightness by an avian visual model) of the wing reflectance measured (A) on the Antero-posterior and (B) the Proximo-distal planes of Morpho wings. The achromatic distances have been measured separately between females (first row of each figure) and males (second row of each figure) of the two species sharing each sympatric location, in order to account for sexual dimorphism. Among each locality, the tested angles are displayed from right to left following the order: [-45°, -30°, -15°, 0°, 15°, 30°, 45°] on the Antero-posterior plane, and [-45°, -30°, -15°, 0°, 15°, 30°, 45°] on the Proximo-distal plane. The dotted line represents the threshold of discrimination of JND=1: visual discrimination is considered possible if the measured the mean achromatic distance (coloured circle) and its confidence interval (coloured vertical line) are superior to the threshold.

**References**

Blandin, P. (2007). *The systematics of the genus Morpho, Fabricius, 1807:(Lepidoptera Nymphalidae, Morphinae)* (Hillside Books).

Vincent, J. F. V., & Wegst, U. G. K. (2004). Design and mechanical properties of insect cuticle. *Arthropod Structure & Development, Arthropod Locomotion Systems: from Biological Materials and Systems to Robotics*, *33*(3), 187‑199. https://doi.org/10.1016/j.asd.2004.05.006
